# Neoadjuvant treatment strategy for locally advanced thoracic esophageal cancer

**DOI:** 10.1002/ags3.12243

**Published:** 2019-03-01

**Authors:** Shuhei Mayanagi, Tomoyuki Irino, Hirofumi Kawakubo, Yuko Kitagawa

**Affiliations:** ^1^ Department of Surgery Keio University School of Medicine Tokyo Japan

**Keywords:** esophagectomy, neoadjuvant chemoradiotherapy, neoadjuvant chemotherapy, perioperative chemotherapy, squamous cell carcinoma

## Abstract

Multimodal treatment combining surgery with chemotherapy and/or radiotherapy is necessary to improve the chances of survival in patients with locally advanced thoracic esophageal cancer. Based on the results of the Japan Clinical Oncology Group 9907 (JCOG9907) trial, neoadjuvant chemotherapy, two courses of cisplatin and 5‐fluorouracil (5‐FU), followed by esophagectomy with D2 lymphadenectomy is the recommended treatment in Japan. Alternatively, neoadjuvant chemoradiotherapy (NACRT) typified by carboplatin and paclitaxel plus concurrent radiotherapy with 41.4 Gy (Chemoradiotherapy for Esophageal Cancer followed by Surgery Study [CROSS]) has shown promising outcomes in some Western countries. Currently, several clinical trials are being conducted within and outside of Japan to confirm the best neoadjuvant treatment regimen. For instance, a three‐arm phase III randomized controlled trial (JCOG1109) is ongoing in Japan. The three arms comprise a doublet regimen (two courses of cisplatin 80 mg/m^2^ day 1 and 5‐FU 800 mg/m^2^ days 1‐5; repeated every 3 weeks) versus a triplet regimen (three courses of docetaxel, 70 mg/m^2^ day 1; cisplatin 70 mg/m^2^ day 1; and 5‐FU 750 mg/m^2^ days 1‐5; repeated every 3 weeks) versus a chemoradiotherapy (CRT) regimen (radiotherapy of 41.4 Gy/23 fractions with two courses of cisplatin 75 mg/m^2^ day 1 and 5‐FU 1000 mg/m^2^ days 1‐4; repeated every 4 weeks). Development of a multimodal strategy for neoadjuvant therapy is expected to receive the continuous focus of research in the hope of achieving better outcomes from treatment of patients with advanced thoracic esophageal cancer.

## 1 INTRODUCTION

Thoracic esophageal cancer is an aggressive malignant tumor with poor prognosis, particularly in the advanced stage. In 2018, esophageal cancer ranked seventh as a common malignancy worldwide, accounting for 572 000 new cases, as well as the sixth most frequent cause of cancer death, accounting for 509 000 deaths.^1^ In Japan, esophageal cancer is also a major cause of mortality that accounts for the seventh largest number of cancer‐associated mortality cases, totaling 11 500 deaths in 2017.^2^ As for histopathological type, squamous cell carcinoma (SCC) accounts for approximately 90% of all cancer cases in Japan.^3^ Surgery, radiotherapy, and chemotherapy have been used as treatments for SCC of the thoracic esophagus. Most patients are diagnosed with locally advanced tumor with lymph node metastasis. Thus, multimodal treatment combining surgery with chemotherapy and radiotherapy seems necessary to improve the chances of survival of such patients.

Recently, neoadjuvant chemotherapy (NAC) followed by esophagectomy with D2 lymphadenectomy has become the recommended treatment in Japan for resectable locally advanced SCC of the thoracic esophagus.^4^, ^5^ However, there exists a discrepancy between Asian and Western countries regarding an appropriate multimodal treatment strategy. From the perspective of local disease control, neoadjuvant chemoradiotherapy (NACRT) was developed in Western countries. This article describes the history and future prospects of the multimodal treatment strategy for locally advanced thoracic cancer in Japan as well as reviewing the current topics in the field.

## 2 TREATMENT STRATEGY TRANSITION IN JAPAN

### 2.1 From postoperative radiotherapy to chemotherapy (JCOG8503 and JCOG9204)

In the 1980s, the standard adjuvant therapy for SCC of the thoracic esophagus in Japan was postoperative radiotherapy. To improve the chances of survival, a randomized control trial (RCT) comparing postoperative radiotherapy (50 Gy) versus two courses of adjuvant chemotherapy using cisplatin (50 mg/m^2^, day 1) and vindesine (3 mg/m^2^, day 1) following esophagectomy was carried out by the Japan Clinical Oncology Group (JCOG) 8503^6^ (Table 1). In this trial, no statistically significant difference was noted between postoperative radiotherapy and adjuvant chemotherapy (5‐year overall survival [OS], 44% vs 42%, respectively). Therefore, after JCOG8503, adjuvant therapeutic strategy in Japan focused on chemotherapy.

**Table 1:** Randomized controlled studies of multimodal therapy for locally advanced thoracic esophageal cancer

Reference		Tumor location	N	SCC (%)	AC (%)	Arm A	Arm B	Outcomes, Arm A vs B		
								Long‐term survival	Surgical outcomes	Response to NAC
Japan										
6	JCOG8503	Esophagus	258			Post‐RT 50 Gy	Post‐CT cisplatin + vindesine	5‐y OS 44% vs 42% (NS)	NA	NA
7	JCOG9204	Esophagus	242	100	0	Surgery alone	Post‐CT cisplatin + 5‐FU	5‐y DFS 45% vs 55% (*P* = 0.037), 5‐y OS 52% vs 61% (*P* = 0.13)	NA	NA
5	JCOG9907	Esophagus	330	100	0	Post‐CT cisplatin + 5‐FU	Pre‐CT cisplatin + 5‐FU	5‐y OS 42% vs 55% (*P* = 0.04)	R0 resection rate 91% vs 96% (*P* = 0.04) Postoperative mortality 0.6% vs 0.6%	Clinical response rate 38% (CR 7%)
Foreign countries										
8, 9	RTOG 8911/USA Intergroup 0113	Esophagus, EGJ	440	47	53	Surgery alone	Pre‐CT cisplatin + 5‐FU	Median OS 16.1 m vs 14.9 m (*P* = 0.53), 2‐y OS 26% vs 23% (*P* = 0.65)	R0 resection rate 59% vs 63% Postoperative mortality 6% vs 6%	Clinical response rate 19% (CR 7%)
10, 11	OEO2	Esophagus, EGJ	802	31	66	Surgery alone	Pre‐CT cisplatin + 5‐FU	5‐y OS 17% vs 23% (*P* = 0.004)	R0 resection rate 54% vs 60% Postoperative morbidity 42% vs 41% Postoperative mortality 10% vs 10%	NA
12	CROSS	Esophagus, EGJ	368	25	75	Surgery alone	Pre‐CRT carboplatin + paclitaxel + 41.4 Gy	5‐y OS 34% vs 47% (*P* = 0.003)	R0 resection rate 69% vs 92% (*P* < 0.001) Postoperative mortality; 4% vs 4%	Pathological CR 29%
(Adenocarcinoma only)										
13	MAGIC	Lower esophagus, EGJ, stomach	503	0	100	Surgery alone	Pre‐ and post‐CT ECF	5‐y OS 23% vs 36% (*P* = 0.009)	R0 resection rate 70% vs 79% (*P* = 0.03) Postoperative morbidity 45% vs 45% Postoperative mortality 6% vs 6%	NA
14	ACCORD07	Lower esophagus, EGJ, stomach	224	0	100	Surgery alone	Pre‐ and post‐CT cisplatin + 5‐FU	5‐y OS 24% vs 38% (*P* = 0.02)	R0 resection rate 74% vs 84% (*P* = 0.04) Postoperative morbidity 19% vs 26% (*P* = 0.24) Postoperative mortality 5% vs 5% (*P* = 0.76)	NA
15	FLOT4‐AIO	EGJ, stomach	300	0	100	Pre‐ and post‐CT ECF/ECX	Pre‐ and post‐CT FLOT	Pathological complete response 6% vs 16% (*P* = 0.02), serious adverse events 40% vs 25%	R0 resection rate 74% vs 85% (*P* = 0.02) Perioperative morbidity 40% vs 25% Postoperative mortality rate 4% vs 2%	Pathological response rate 23% vs 37% (*P* = 0.02) (CR 6% vs 16%, *P* = 0.02)

AC, adenocarcinoma; CR, complete response; CROSS, Chemoradiotherapy for Esophageal Cancer followed by Surgery Study; CRT, chemoradiotherapy; CT, chemotherapy; DFS, disease‐free survival; ECF, epirubicin + cisplatin + 5‐fluorouracil; ECX, epirubicin + cisplatin + capecitabine; EGJ, esophagogastric junction; FLOT, docetaxel + oxaliplatin + leucovorin + fluorouracil; MAGIC, Medical Research Council Adjuvant Gastric Infusional Chemotherapy; NA, not available; NAC, neoadjuvant chemotherapy; NS, not statistically significant at level of 0.05; OS, overall survival; RT, radiotherapy; SCC, squamous cell carcinoma.

Changing the adjuvant chemotherapy regimen from vindesine to 5‐fluorouracil (5‐FU), a phase III RCT JCOG9204 in which the outcomes of surgery alone were compared with that of surgery plus adjuvant chemotherapy using two courses of cisplatin (80 mg/m^2^, day 1) and 5‐FU (800 mg/m^2^, days 1‐5) was conducted after JCOG 8503.^7^ In the adjuvant cisplatin and 5‐FU groups, improvement in the 5‐year recurrent‐free survival for the surgery‐alone group (55% and 45%, *P* = 0.037) was noted. From the results of JCOG9204, a cisplatin and 5‐FU regimen was established as standard for adjuvant chemotherapy in Japan. Moreover, in the JCOG trials from the 1980s to the 1990s, the 5‐year OS increased by >10%. From the 1980s, quality improvement in D2 lymph node dissection, including the upper mediastinal nodes, was regarded as an important factor.

### 2.2 Neoadjuvant chemotherapy using 5‐FU and cisplatin followed by surgery (JCOG9907)

Furthermore, to evaluate the optimal perioperative timing of adjuvant chemotherapy (before or after surgery), the JCOG9907 trial was conducted.^5^ In this trial, patients with clinical stage II/III SCC of the thoracic esophagus, according to the Japanese Classification of Esophageal Cancer, were randomly assigned to the group (n = 164) with two courses of cisplatin (80 mg/m^2^, day 1) and 5‐FU (800 mg/m^2^, days 1‐5) NAC followed by surgery or to the group with an identical regimen of postoperative chemotherapy (n = 166). The 5‐year OS in the NAC group was significantly superior to that of the postoperative chemotherapy group (55% vs 42%, respectively) (hazard ratio [HR] 0.73, *P* = 0.04).

Meanwhile, chemoradiotherapy (CRT) with a radiation dose of >50 Gy was another option for definitive therapy for thoracic esophageal cancer. In Japan, the single‐arm phase II trial JCOG9906 with concurrent use of irradiation, totaling 60 Gy, cisplatin and 5‐FU, showed a 5‐year survival rate of 37%.^16^

No RCT in Japan compared NAC followed by surgery with definitive CRT; therefore, from the results of JCOG trials 9907/9906 and several observational studies, NAC followed by esophagectomy with D2 lymphadenectomy is presently recommended in the Japanese guidelines. Thus, the first choice for physiologically fit patients with locally advanced SCC of the thoracic esophagus is NAC using cisplatin and 5‐FU, followed by surgery. Furthermore, postoperative adjuvant chemotherapy is considered on the basis of pathological findings, particularly in cases of positive metastasis in the lymph node if a patient cannot receive chemotherapy in the neoadjuvant setting for any reason.

### 2.3 Definitive CRT followed by salvage treatment (JCOG0909)

In patients selected for definitive concurrent CRT because of their general condition or patient's preference, salvage surgery is a possible therapeutic option for recurrent or residual cancer of non‐responders. The single‐arm phase II trial (JCOG0909) was planned to verify the efficacy and safety of CRT strategy for locally advanced SCC of the esophagus, followed by salvage surgery or endoscopic resection for recurrent or residual tumor. In JCOG0909, CRT comprises cisplatin (75 mg/m^2^, days 1, 29), 5‐FU (1000 mg/m^2^, days 1‐4, 29‐32), and radiotherapy (50.4 Gy/28 fractions), followed by two additional courses of chemotherapy with cisplatin and 5‐FU. Salvage therapy, including surgery and/or endoscopic resection, is applied when the patient has residual or recurrent tumor after definitive CRT. After definitive CRT, complete response was achieved in 59% of all cases, and the 3‐year OS rate was 74%. Salvage endoscopic resection and surgery were carried out in five patients (5%) and 25 patients (27%), respectively.^17^ Although the sample size in the single‐arm phase II study was small, these results suggest that definitive CRT could be a promising option as an initial treatment for patients with a strong preference for sparing the esophagus.

## 3 CURRENT WORLDWIDE TRENDS

It is essential to pay attention to the histopathological differences in esophageal cancer between patients from East Asia, including Japan, and Western countries. More than 60% of all patients in Western countries have adenocarcinoma (AC) of the esophagus or esophagogastric junction (EGJ). Thus, clinical trials are planned to focus on patients with AC. Moreover, considering surgery, upper mediastinal lymph node dissection is usually omitted in Ivor‐Lewis esophagectomy. In response, aggressive neoadjuvant treatment, including CRT, has been developed for local disease control.

### 3.1 Perioperative chemotherapy

In the 1990s, unlike the Japanese clinical trials, the superiority of surgery plus perioperative chemotherapy using 5‐FU and cisplatin was not verified in the RTOG 8911/USA Intergroup 0113 trial.^8^, ^9^ Alternatively, the OEO2 trial conducted by the Medical Research Council in the UK suggested that preoperative chemotherapy using 5‐FU and cisplatin in comparison with surgery alone had a significant survival benefit with an HR of 0.84 (5‐year OS 23% vs 17%).^10^, ^11^ In the OEO2 trial, the treatment effect was consistent in both AC and SCC.

The Medical Research Council Adjuvant Gastric Infusional Chemotherapy (MAGIC) trial mainly focused on gastric AC, although it included part of the lower esophageal and esophagogastric AC. This trial reported that perioperative treatment, that is, three preoperative and three postoperative cycles of chemotherapy using a triplet of epirubicin, cisplatin and 5‐FU (ECF) as compared with surgery alone significantly improved survival (5‐year OS: 36% vs 23%).^13^ Regarding perioperative chemotherapy, in the French ACCORD‐07, the use of a perioperative combination of cisplatin and FU significantly improved OS in patients with esophageal, EGJ or gastric AC as compared to surgery alone (5‐year OS 38% vs 24%).^14^

Furthermore, the FLOT4 trial, which compared EGJ and gastric AC with docetaxel‐based triplet chemotherapy (including four preoperative and four postoperative 2‐week cycles of docetaxel, oxaliplatin, leucovorin and FU; FLOT) versus an anthracycline‐based triplet chemotherapy (including preoperative and postoperative 3‐week cycles of epirubicin, cisplatin, and FU or capecitabine), conducted in Germany, and the phase II part of this trial showed higher proportions of patients achieving pathological complete regression and fewer adverse events in the FLOT group.^15^ In the phase III part, 3‐year OS in the FLOT group was significantly superior to that in the anthracycline‐based triplet chemotherapy group (57% vs 48%, respectively; HR 0.75, *P* = 0.004).

### 3.2 Neoadjuvant chemoradiotherapy

Good outcomes have been achieved from the perspective of local disease control using NACRT. The Trans‐Tasman Radiation Oncology Group reported that preoperative CRT of 35 Gy with cisplatin and FU did not significantly improve OS in comparison to that with surgery alone. However, subgroup analysis in that study showed that patients with SCC had better progression‐free survival than patients with AC.^18^

In the Chemoradiotherapy for Esophageal Cancer followed by Surgery Study (CROSS), patients with esophageal or EGJ cancer were randomly assigned to the surgery‐alone group or to the group with weekly carboplatin and paclitaxel and concurrent radiotherapy (41.4 Gy) followed by surgery.^12^ Pathological complete response was achieved in 29% of the patients who underwent resection after CRT. Postoperative complications were similar in the two treatment groups. Median OS was 49 months in the CRT group versus 24 months in the surgery‐alone group (HR, 0.657; *P* = 0.003, 5‐year OS, 47% vs 34%).

Based on the results of these RCT and meta‐analyses, the European Society for Medical Oncology (ESMO) guidelines and the National Comprehensive Cancer Network (NCCN) guidelines have classified the treatment strategies according to histopathological type. The ESMO guidelines^19^ describe that patients with locally advanced SCC of the esophagus benefit from preoperative chemotherapy or are most likely to benefit to a greater extent from NACRT, with higher rates of complete tumor resection and better local tumor control and survival. The CROSS regimen can be recommended as a contemporary standard of care. NACRT is also a recommended approach for advanced SCC of the esophagus according to the NCCN guidelines (version 2.2018), and both the CROSS and FOLFOX^20^ regimens are preferred. In the setting of definitive CRT, the PRODIGE5/ACCORD17 trial showed that the use of oxaliplatin is safer and more convenient, with fewer cases of sudden death, than cisplatin.^20^ The PROTECT trial is an ongoing randomized phase II trial comparing two NACRT regimens (CROSS vs FOLFOX) in resectable esophageal and EGJ cancers of SCC or AC.^21^

## 4 FUTURE PERSPECTIVES: WHAT IS THE OPTIMAL NEOADJUVANT THERAPY – NAC OR NACRT?

According to the results of JCOG9907, the preoperative setting offers the advantage of dose intensity in chemotherapy in comparison with that in the postoperative setting. Based on the subgroup analysis in the JCOG9907 trial, only a small benefit was noted in OS between the preoperative and postoperative groups, particularly in patients with stage III esophageal cancer. A more aggressive regimen than with cisplatin and 5‐FU is required for advanced esophageal cancer treatment. Addition of taxan to platinum and FU triplet regimen has been developed for treating head and neck cancer or gastric cancer. In esophageal cancer, a combination of docetaxel, cisplatin, and 5‐FU in the form of NAC has shown promising results in several phase II studies.^22^, ^23^, ^24^, ^25^ Although hematological toxicity, especially neutropenia, was common in 80% of all cases during NAC with the triplet regimen, pathological complete response rate of 7%‐13% after NAC with the triplet regimen was relatively higher than that after NAC with the doublet regimen of 5‐FU and cisplatin. Therefore, the triplet regimen may deliver a strong antitumor effect and potentially improve long‐term outcomes. However, as previously indicated, NACRT typified by the CROSS trial has revived interest as a promising treatment. It is thus notable that in the CROSS trial, a positive effect of CRT was remarkable in patients with SCC.

There is only one RCT with a relatively large sample size comparing NAC with NACRT followed by surgery for esophageal or esophagogastric cancer (NeoRes trial).^26^ In this trial, three cycles of cisplatin and FU were given in both arms, whereas 40 Gy of concomitant radiotherapy was included in the NACRT arm. Higher histological complete response was achieved in 28% of cases after NACRT versus in 9% of cases after NAC (*P* = 0.001). However, the 3‐year OS was similar between both arms (49% in chemotherapy arm vs 47% in CRT arm, *P* = 0.77). Presently, no specific evidence has been obtained from the comparison between perioperative chemotherapy versus NACRT.

In practice, NAC with 5‐FU and cisplatin has been the standard of care for patients with locally advanced esophageal cancer in Japan. In cases with multiple lymph node metastases spreading three fields from the neck to the abdomen or enlarged lymph node metastasis, it may be more appropriate to select NAC with a triplet regimen such as the combination of docetaxel, cisplatin, and 5‐FU than with NACRT because the irradiation field would then be extremely wide in the NACRT setting. However, for borderline resectable cases suspected of adjacent organ invasion, NACRT may be considered with an emphasis on local disease control.

**Figure 1:**
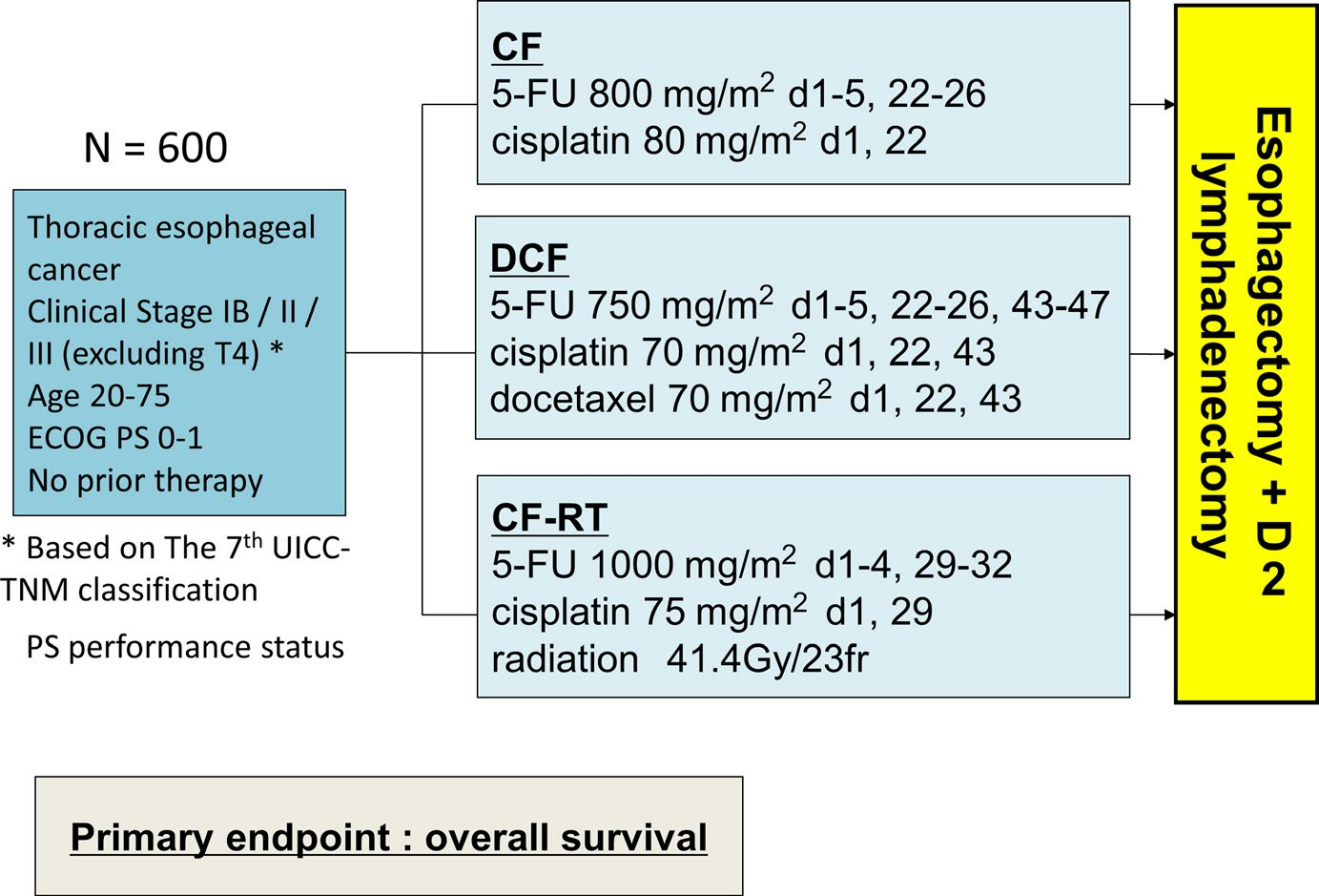
JCOG1109 study design. Patients with locally advanced esophageal cancer were randomized to any of the three arms consisting of arm A (preoperative CF), arm B (preoperative DCF), and arm C (preoperative CF‐RT), followed by surgery. Primary endpoint was overall survival. CF, cisplatin plus 5‐fluorouracil (5‐FU); CF‐RT, radiotherapy with CF; DCF, docetaxel, cisplatin plus 5‐FU; JCOG, Japan Clinical Oncology Group

Presently, in Japan, a three‐arm phase III RCT (JCOG1109) is ongoing to confirm the best neoadjuvant treatment regimen for SCC (Figure 1). The three arms comprise a doublet regimen (including two courses of cisplatin 80 mg/m^2^ day 1 and 5‐FU 800 mg/m^2^ days 1‐5; repeated every 3 weeks) versus a triplet regimen (including three courses of docetaxel, 70 mg/m^2^ day 1; cisplatin 70 mg/m^2^ day 1; and 5‐FU 750 mg/m^2^ days 1‐5; repeated every 3 weeks) versus a CRT regimen (including radiation of 41.4 Gy/23 fractions with two courses of cisplatin 75 mg/m^2^ day 1 and 5‐FU 1000 mg/m^2^ days 1‐4; repeated every 4 weeks).^27^

For AC of the esophagus and EGJ, two large‐scale RCT comparing perioperative chemotherapy with NACRT followed by surgery have also been started in other countries. One of them is the ESOPEC trial that compares the CROSS protocol (41.4 Gy plus carboplatin/paclitaxel) versus FLOT protocol (5‐FU/leucovorin/oxaliplatin/docetaxel),^28^ and the other is the Neo‐AEGIS trial comparing the CROSS protocol versus the modified MAGIC protocol (epirubicin/cisplatin or oxaliplatin/5‐FU or capecitabine).^29^ The optimal adjuvant strategy for esophageal and EGJ cancer is expected to be derived from these RCT.

In addition, promising results relating to anti‐programmed cell death protein 1 antibody for treatment‐refractory advanced esophageal cancer have been reported in a phase II trial.^30^ Immune checkpoint inhibitors and molecular targeting drugs are also expected to function as an option for adjuvant therapy along with chemotherapeutic drugs for esophageal cancer.

In Europe, a new organ‐sparing approach called active surveillance has been proposed based on the high pathological complete response rate (49% for SCC and 23% for AC) after NACRT of the CROSS trial. In this organ‐sparing strategy, patients with clinical complete response after NACRT undergo frequent diagnostic evaluations, including endoscopy, with fine‐needle aspiration of suspected lymph nodes and ^18^F‐fluorodeoxyglucose (FDG)‐positron emission tomography (PET)/computed tomography (CT). An esophagectomy is carried out only in patients with a proven or high suspicion of locoregional regrowth. The effectiveness of this active surveillance as compared to standard surgery has been assessed in the Surgery As Needed approach in Oesophageal cancer patients (SANO) trial.^31^ As reported from the JCOG0909 trial, in Japan, CRT followed by salvage surgery can provide a promising therapeutic strategy. In the future, comparison of NACRT plus planned surgery with the esophagus‐sparing treatment approach may be necessary not only for survival benefit but also for quality of life.

In conclusion, developmental research in a multimodal strategy for neoadjuvant therapy is expected to be continued in the hope of achieving better outcomes for treatment of patients with locally advanced thoracic esophageal cancer.

## DISCLOSURE

Conflicts of Interest: Y. Kitagawa received designated donation from Yakult Honsha Co., Ltd, Kyowa Hakko Kirin Co., Ltd, Takeda Pharmaceutical Co., Ltd, Chugai Pharmaceutical Co., Ltd, and ONO Pharmaceutical Co., Ltd. His institution has endowed chairs of Chugai Pharmaceutical Co., Ltd and ONO Pharmaceutical Co., Ltd.

## References

[1] Bray F , Ferlay J , Soerjomataram I , et al. Global cancer statistics 2018: GLOBOCAN estimates of incidence and mortality worldwide for 36 cancers in 185 countries. CA Cancer J Clin. 2018;68(6):394–424. 30207593 10.3322/caac.21492

[2] Cancer Information Service NCC, Japan . Cancer Registry and Statistics. In. 2017.

[3] Tachimori Y , Ozawa S , Numasaki H , et al. Comprehensive Registry of Esophageal Cancer in Japan, 2010. Esophagus. 2017;14:189–214. 28725168 10.1007/s10388-017-0578-4 PMC5486463

[4] Japan Esophageal Society . Guidelines for Diagnosis and Treatment of Carcinoma of the Esophagus 2017. Tokyo: Kanehara 2017.

[5] Ando N , Kato H , Igaki H , et al. A randomized trial comparing postoperative adjuvant chemotherapy with cisplatin and 5‐fluorouracil versus preoperative chemotherapy for localized advanced squamous cell carcinoma of the thoracic esophagus (JCOG9907). Ann Surg Oncol. 2012;19:68–74. 21879261 10.1245/s10434-011-2049-9

[6] Japanese Esophageal Oncology Group . A comparison of chemotherapy and radiotherapy as adjuvant treatment to surgery for esophageal carcinoma. Chest. 1993;104:203–275. 8325071 10.1378/chest.104.1.203

[7] Ando N , Iizuka T , Ide H , et al. Surgery plus chemotherapy compared with surgery alone for localized squamous cell carcinoma of the thoracic esophagus: a Japan Clinical Oncology Group Study—JCOG9204. J Clin Oncol. 2003;21:4592–6. 14673047 10.1200/JCO.2003.12.095

[8] Kelsen DP , Ginsberg R , Pajak TF , et al. Chemotherapy followed by surgery compared with surgery alone for localized esophageal cancer. N Engl J Med. 1998;339:1979–84. 9869669 10.1056/NEJM199812313392704

[9] Kelsen DP , Winter KA , Gunderson LL , et al. Long‐term results of RTOG Trial 8911 (USA Intergroup 113): a random assignment trial comparison of chemotherapy followed by surgery compared with surgery alone for esophageal cancer. J Clin Oncol. 2007;25:3719–25. 17704421 10.1200/JCO.2006.10.4760

[10] Medical Research Council Oesophageal Cancer Working Group . Surgical resection with or without preoperative chemotherapy in oesophageal cancer: a randomised controlled trial. Lancet. 2002;359:1727–33. 12049861 10.1016/S0140-6736(02)08651-8

[11] Allum WH , Stenning SP , Bancewicz J , et al. Long‐term results of a randomized trial of surgery with or without preoperative chemotherapy in esophageal cancer. J Clin Oncol. 2009;27:5062–275. 19770374 10.1200/JCO.2009.22.2083

[12] van Hagen P , Hulshof MC , van Lanschot JJ , et al. Preoperative chemoradiotherapy for esophageal or junctional cancer. N Engl J Med. 2012;366:2074–84. 22646630 10.1056/NEJMoa1112088

[13] Cunningham D , Allum WH , Stenning SP , et al. Perioperative chemotherapy versus surgery alone for resectable gastroesophageal cancer. N Engl J Med. 2006;355:11–20. 16822992 10.1056/NEJMoa055531

[14] Ychou M , Boige V , Pignon J‐P , et al. Perioperative chemotherapy compared with surgery alone for resectable gastroesophageal adenocarcinoma: an FNCLCC and FFCD multicenter phase III trial. J Clin Oncol. 2011;29:1715–21. 21444866 10.1200/JCO.2010.33.0597

[15] Al‐Batran S‐E , Hofheinz RD , Pauligk C , et al. Histopathological regression after neoadjuvant docetaxel, oxaliplatin, fluorouracil, and leucovorin versus epirubicin, cisplatin, and fluorouracil or capecitabine in patients with resectable gastric or gastro‐oesophageal junction adenocarcinoma (FLOT4‐AIO): results from the phase 2 part of a multicentre, open‐label, randomised phase 2/3 trial. Lancet Oncol. 2016;17:1697–708. 27776843 10.1016/S1470-2045(16)30531-9

[16] Kato K , Muro K , Minashi K , et al. Phase II study of chemoradiotherapy with 5‐fluorouracil and cisplatin for Stage II‐III esophageal squamous cell carcinoma: JCOG trial (JCOG 9906). Int J Radiat Oncol Biol Phys. 2011;81:684–90. 20932658 10.1016/j.ijrobp.2010.06.033

[17] Ito Y , Ogawa G , Kato K , et al. A single‐arm confirmatory study of definitive chemoradiotherapy including salvage treatment in patients with clinical stage II/III esophageal carcinoma (JCOG0909). J Clin Oncol. 2018;36:(suppl):abstr 4051.

[18] Burmeister BH , Smithers BM , Gebski V , et al. Surgery alone versus chemoradiotherapy followed by surgery for resectable cancer of the oesophagus: a randomised controlled phase III trial. Lancet Oncol. 2005;6:659–68. 16129366 10.1016/S1470-2045(05)70288-6

[19] Lordick F , Mariette C , Haustermans K , et al. Oesophageal cancer: ESMO Clinical Practice Guidelines for diagnosis, treatment and follow‐up. Ann Oncol. 2016;27:v50–275. 27664261 10.1093/annonc/mdw329

[20] Conroy T , Galais M‐P , Raoul J‐L , et al. Definitive chemoradiotherapy with FOLFOX versus fluorouracil and cisplatin in patients with oesophageal cancer (PRODIGE5/ACCORD17): final results of a randomised, phase 2/3 trial. Lancet Oncol. 2014;15:305–14. 24556041 10.1016/S1470-2045(14)70028-2

[21] Messager M , Mirabel X , Tresch E , et al. Preoperative chemoradiation with paclitaxel‐carboplatin or with fluorouracil‐oxaliplatin‐folinic acid (FOLFOX) for resectable esophageal and junctional cancer: the PROTECT‐1402, randomized phase 2 trial. BMC Cancer. 2016;16:318 10.1186/s12885-016-2335-9. 27194176 PMC4872363

[22] Hara H , Tahara M , Daiko H , et al. Phase II feasibility study of preoperative chemotherapy with docetaxel, cisplatin, and fluorouracil for esophageal squamous cell carcinoma. Cancer Sci. 2013;104:1455–60. 23991649 10.1111/cas.12274 PMC7654256

[23] Watanabe M , Baba Y , Yoshida N , et al. Outcomes of preoperative chemotherapy with docetaxel, cisplatin, and 5‐fluorouracil followed by esophagectomy in patients with resectable node‐positive esophageal cancer. Ann Surg Oncol. 2014;21:2838–44. 24715216 10.1245/s10434-014-3684-8

[24] Sudarshan M , Alcindor T , Ades S , et al. Survival and recurrence patterns after neoadjuvant docetaxel, cisplatin, and 5‐fluorouracil (DCF) for locally advanced esophagogastric adenocarcinoma. Ann Surg Oncol. 2015;22:324–30. 25023544 10.1245/s10434-014-3875-3

[25] Ui T , Fujii H , Hosoya Y , et al. Comparison of preoperative chemotherapy using docetaxel, cisplatin and fluorouracil with cisplatin and fluorouracil in patients with advanced carcinoma of the thoracic esophagus. Dis Esophagus. 2015;28:180–275. 24529073 10.1111/dote.12187

[26] Klevebro F , Alexandersson von Dobeln G , Wang N , et al. A randomized clinical trial of neoadjuvant chemotherapy versus neoadjuvant chemoradiotherapy for cancer of the oesophagus or gastro‐oesophageal junction. Ann Oncol. 2016;27:660–275. 26782957 10.1093/annonc/mdw010

[27] Nakamura K , Kato K , Igaki H , et al. Three‐arm phase III trial comparing cisplatin plus 5‐FU (CF) versus docetaxel, cisplatin plus 5‐FU (DCF) versus radiotherapy with CF (CF‐RT) as preoperative therapy for locally advanced esophageal cancer (JCOG1109, NExT study). Jpn J Clin Oncol. 2013;43:752–5. 23625063 10.1093/jjco/hyt061

[28] Hoeppner J , Lordick F , Brunner T , et al. ESOPEC: prospective randomized controlled multicenter phase III trial comparing perioperative chemotherapy (FLOT protocol) to neoadjuvant chemoradiation (CROSS protocol) in patients with adenocarcinoma of the esophagus (NCT02509286). BMC Cancer. 2016;16:503. 27435280 10.1186/s12885-016-2564-y PMC4952147

[29] Reynolds JV , Preston SR , O'Neill B , et al. ICORG 10‐14: NEOadjuvant trial in Adenocarcinoma of the oEsophagus and oesophagoGastric junction International Study (Neo‐AEGIS). BMC Cancer. 2017;17:401. 28578652 10.1186/s12885-017-3386-2 PMC5457631

[30] Kudo T , Hamamoto Y , Kato K , et al. Nivolumab treatment for oesophageal squamous‐cell carcinoma: an open‐label, multicentre, phase 2 trial. Lancet Oncol. 2017;18:631–9. 28314688 10.1016/S1470-2045(17)30181-X

[31] Noordman BJ , Wijnhoven BPL , Lagarde SM , et al. Neoadjuvant chemoradiotherapy plus surgery versus active surveillance for oesophageal cancer: a stepped‐wedge cluster randomised trial. BMC Cancer. 2018;18:142. 29409469 10.1186/s12885-018-4034-1 PMC5801846

